# Preparation and Properties of YP50S/TPOSS/Si-MMT Synergistically Modified Cyanate Ester Resin Carrier Film for High-Frequency and High-Speed Copper-Clad Laminate

**DOI:** 10.3390/ma19102159

**Published:** 2026-05-21

**Authors:** Jiayu Gao, Qichen Yin, Junfeng Qiang

**Affiliations:** 1 School of Materials Science and Engineering, Xi’an University of Science and Technology, Xi’an 710021, China; gaojiayu0914@163.com; 2 School of Chemistry and Chemical Engineering, Xi’an Jiaotong University, Xi’an 710049, China

**Keywords:** cyanate ester resin, YP50S, TPOSS, Si-MMT, basalt fiber, copper clad laminate

## Abstract

**Highlights:**

1. After synergistic modification with 3% Si-MMT, the dielectric constant of the composite decreases from approximately 3.4 to 3.3 at 1 × 10^7^ Hz, while maintaining a low dielectric loss.
2. The modified system containing 10% YP50S, 0.5% TPOSS, and 3% Si-MMT exhibits optimal comprehensive performance for high-frequency and high-speed copper-clad laminates (CCL).
3. The synergistic catalysis of YP50S and TPOSS reduces the curing temperature of cyanate ester resins by 89 °C, addressing the bottlenecks of high curing temperature and poor toughness, which is conducive to energy saving, consumption reduction, and industrial production.
4. With the addition of 3% Si-MMT, the flexural strength reaches 540.5 MPa, and the 5% weight-loss temperature rises to 400 °C, achieving simultaneous improvement in mechanical properties and thermal stability to meet the high reliability and heat resistance requirements of high-frequency CCL.
5. Excellent dielectric properties (low dielectric constant of 3.3 and low loss) are achieved, which can effectively reduce high-frequency signal transmission delay and insertion loss, supporting the development of 5G/6G communications.

**Abstract:**

To meet the demands for high-frequency, high-speed copper-clad laminates characterized by low dielectric constant, low loss, high heat resistance, and good mechanical performance, this study employs cyanate ester resin (CE) as the matrix. Additionally, polyphenylene oxide resin (YP50S), TPOSS, and silicon-intercalated montmorillonite (Si-MMT) are introduced for synergistic modification. Basalt fiber is further incorporated to prepare composite carrier films. The results demonstrate that YP50S significantly accelerates the curing of CE, resulting in a reduction in the curing peak temperature by 89 °C at a concentration of 10 wt%. TPOSS further amplifies this effect. Si-MMT markedly enhances the overall properties, with 3 wt% yielding optimal performance. The dielectric constant decreases to 3.3 at 1 × 10^7^ Hz while maintaining a low dielectric loss. This strategy effectively enhances the overall performance of CE-based composites.

## 1. Introduction

With the development of 5G/6G communication, vehicle-mounted radar and high-speed interconnection, high-frequency and high-speed copper-clad laminate (CCL) must meet the requirements of low dielectric constant (Dk) and low dielectric loss (Df) to reduce transmission delay and insertion loss. In addition, it must also take into account comprehensive indicators such as thermal stability, hygroscopicity, dimensional stability and copper foil bonding reliability [1,2,3,4]. After curing, cyanate ester resin (CE) forms a triazine ring crosslinking network [5], which has excellent dielectric properties, heat and moisture resistance and high glass transition temperature. It is an important candidate system for high-frequency and high-speed substrates. However, due to its high crosslinking density, strong structural rigidity, large brittleness of the cured product and limited process window, it is still necessary to achieve performance balance through structure/formula regulation [6]. Studies have shown that the construction of an interpenetrating network by blending with thermosetting resins such as benzoxazine and epoxy can improve toughness and heat resistance while maintaining low dielectric parameters [7,8]. For example, the CE/BZ/EP multi-component system can obtain lower Df and higher thermal stability at a suitable ratio, and significantly reduce the moisture absorption rate [9]. At the same time, the introduction of low polar aromatic ether components (such as PPO) is one of the effective ways to further reduce the loss, but its impact on the curing network, interface bonding and comprehensive reliability still needs to be weighed in combination with the system design [10]. YP50S is a commercial phenoxy resin, which belongs to the family of polyphenylene oxide (PPO) derivatives; therefore, it inherits the low-polarity characteristic of PPO while providing additional hydroxyl groups that are beneficial for toughening and catalytic curing. On the other hand, phenoxy resin has advantages in toughening, bonding and process adaptation due to its ether bond and hydroxyl structure [11]. The development of special phenoxy resin for copper-clad laminate/packaging plate also shows the potential of synergistic improvement of low Dk/Df and heat resistance [12,13]. In addition, layered nanofillers (such as intercalated modified montmorillonite) can regulate segment motion and microstructure through interface constraints and barrier effects, thus providing a new means to improve thermal stability, moisture resistance and dielectric stability [14]. Based on the above background, this paper uses bisphenol A cyanate ester (CE) as the main resin, introduces phenoxy resin (YP50S) and trisilanol phenyl polyhedral oligomeric silsesquioxane (TPOSS) to synergistically regulate the curing behavior and network structure, and adds silicon intercalated montmorillonite to construct a micro-nano interface structure. At the same time, basalt fiber was used as the reinforced base [15]. The curing characteristics, dielectric properties, thermal stability and interfacial bonding properties of the composite system were systematically studied to provide reference for the design of low loss, high reliability and high-frequency copper-clad laminate substrate. It is worth noting that ultrasonic-assisted dispersion techniques, as reported by Ganguly et al. [16], could further improve the nanoscale dispersion of Si-MMT in future studies.

## 2. Materials and Methods

### 2.1. Main Raw Materials

**Figure 1:**
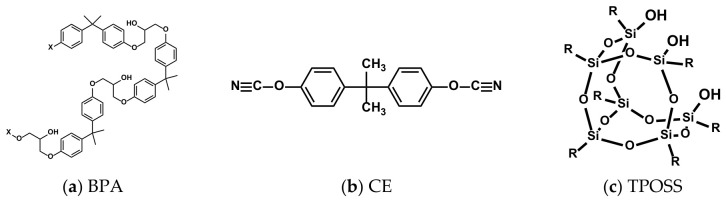
Structural formula of BPA and CE and TPOSS.

Basalt fiber (BF) cloth with an areal density of 200 g/m^2^ has both warp and weft yarn densities of 5 yarns per centimeter, and its average yarn spacing is 2 mm (Haining Anjie Composite Materials Co., Ltd., Jiaxing, Zhejiang Province, China). Bisphenol A dicyanate (CE) was obtained from Yangzhou Tianqi New Material Co., Ltd., Yangzhou, Jiangsu Province, China. Phenoxy resin (YP50S) was prepared by Beijing University of Chemical Technology, Beijing, China. Montmorillonite (MMT), specifically silicon-intercalated modified montmorillonite (Si-MMT, 1250 mesh), was supplied by Yanbo Mineral Products Processing Company, Zhangjiakou, Hebei Province, China. Trisilanol phenyl polyhedral oligomeric silsesquioxane (TPOSS) was self-made (TPOSS was synthesized via the corner-capping method. Typically, phenyltrimethoxysilane was hydrolyzed and condensed under acidic conditions to form an incompletely condensed silsesquioxane intermediate, which was then reacted with additional phenyltrimethoxysilane in the presence of a base catalyst (tetramethylammonium hydroxide) to yield the fully condensed trisilanol phenyl POSS. The product was purified by recrystallization from methanol/acetone mixture and characterized by FTIR and ^1^H NMR [17]). Figure 1 shows the structural formulas of BPA, CE, and TPOSS.

### 2.2. Instruments and Equipment

Differential scanning calorimeter (DSC), DSC21, METTLER TOLEDO, Greifensee, Switzerland (test conditions: temperature range 30–300 °C, heating rate 10 °C/min, N_2_ flow rate 50 mL/min); rheometer, MCR302e, Anton Paar (Shanghai) Trading Co., Ltd., Shanghai, China (test conditions: temperature range 80–200 °C, heating rate 10 °C/min, Oscillating shear mode); electronic universal testing machine, C45.105, Zhejiang Zhirou Technology Co., Ltd., Jiaxing, Zhejiang Province, China (five specimens were tested for each formulation), results are reported as mean ± standard deviation (SD).; thermogravimetric analyzer, TGA2, Niche Co., Ltd., Shanghai, China, Germany (test conditions: temperature range 30–800 °C, heating rate 10 °C/min, N_2_ flow rate 50 mL/min); dielectric spectrometer, Concept 80, Beijing Huidexin Technology Co., Ltd., Beijing, China (test frequency range: 1 Hz to 10^7^ Hz). All measurements were performed at least 3 times or more to ensure repeatability.

### 2.3. Sample Preparation

#### 2.3.1. Preparation of Toughening Modified Cyanate Ester

Table 1 is the formula of modified cyanate ester resin system.

(1) Toughening modification of cyanate ester resin by phenoxy resin

CE and YP50S were reacted by thermal mixing. CE was added to a 250 mL three-necked flask and placed in an oil bath at 160 °C. After hot melting, YP50S was added. The mixture was allowed to react for 30 min, and bubbles were removed under vacuum for 30 min to obtain a phenoxy resin-modified cyanate ester resin.

(2) Phenoxy resin-polyhedral oligomeric silsesquioxane toughening-modified cyanate ester resin

A certain amount of CE resin was added to a three-necked flask, and the oil bath was heated to 140 °C. When the CE was completely melted, YP50S was added and mixed evenly, and a small amount of TPOSS was added as a reaction catalyst. The mixture was then stirred for 10 min, and bubbles were removed under vacuum for 30 min to obtain a toughened modified cyanate ester resin.

(3) Phenoxy resin-polyhedral oligomeric silsesquioxane-Si-MMT toughening-modified cyanate ester resin

YP50S with a mass fraction of 10% was mixed with CE resin and added into a three-necked flask. The temperature was increased to 140 °C. When the CE was completely melted, mechanical stirring was started. After CE and YP50S had reacted for 30 min, the temperature was reduced to 120 °C. Si-MMT with mass fractions of 1%, 2%, and 3% was added respectively. After mixing evenly, TPOSS with a mass fraction of 0.5% was added as a reaction catalyst, and the mixture was stirred for 10 min. Bubbles were removed under vacuum for 30 min to obtain a phenoxy resin/polyhedral oligomeric silsesquioxane/Si-MMT modified cyanate ester resin.

#### 2.3.2. Preparation of Carrier Film

Twenty pieces of basalt fiber fabric (each 150 mm × 150 mm, areal density 200 g/m^2^) were used. Each piece was impregnated with 3 g of the polymer resin and then pressed at 110 °C under a pressure of 1 MPa for 5 s (heating rate: 5 °C/min) to form a single-layer prepreg. After this rapid pressing, the prepreg was immediately removed. Subsequently, the twenty prepreg layers were stacked together and consolidated under a pressure of 2 MPa in a standard laboratory atmosphere (no vacuum) following the curing cycle: 140 °C/2 h + 180 °C/2 h + 210 °C/2 h + 240 °C/2 h, with a heating rate of 5 °C/min between each step.

## 3. Results and Discussion

### 3.1. Analysis of Curing Behavior of Different Toughening Modified Cyanate Ester Resin

**Figure 2:**
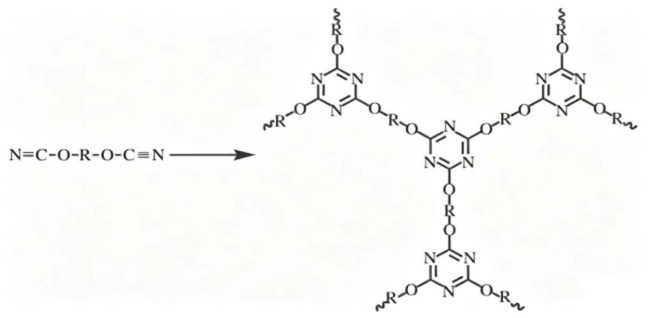
Reaction equation of self-polymerization of CE.

The curing behavior of YP50S, TPOSS, and Si-MMT modified CE was measured by DSC, and the curing process of each modified resin system was determined. The curing process directly affects the final properties of the resin. Too low a curing temperature and too long a curing time are not conducive to industrial production and application. If the curing temperature is too high, explosive polymerization occurs, and insufficient time for constructing the polymer network may lead to defects in the cured product. Therefore, selecting an appropriate curing process is very important. Differential scanning calorimetry can determine the optimal curing process for the resin, and the gradient curing method is generally used. The curing reaction of CE is relatively simple, typically showing a sharp melting endothermic peak at low temperatures and a broad curing exothermic peak at high temperatures [18]. Figure 2 shows the self-polymerization reaction equation of CE [19]. As shown in Figure 2, under external heating or an internal catalyst, CE monomers can undergo self-polymerization crosslinking to form a highly symmetrical three-dimensional triazine ring network [20]. This structure endows the resin with excellent mechanical properties and high-temperature resistance, but its high curing temperature limits its application range.

It can be seen from Table 2 that with increasing YP50S mass fraction, the melting endothermic peak of the CE/YP50S blends gradually weakened, indicating that the flexible long-chain structure of YP50S can reduce the crystallinity of CE, which is beneficial for the processing of CE resin. At the same time, the curing exothermic peak of the CE/YP50S blends shifted significantly to lower temperatures, and the peak temperature gradually decreased, indicating that phenoxy resin has a significant catalytic effect on the curing of CE resin. This is mainly because, during the curing process, the hydroxyl groups in the YP50S molecule can promote the cyclotrimerization reaction of cyanate ester monomers through proton transfer, leading to the formation of a triazine ring structure [21,22]. When the mass fraction of YP50S is 10 wt%, the catalytic effect is remarkable, and the curing peak temperature is 89 °C lower than that of pure CE resin. However, as the mass fraction of YP50S continues to increase, the curing peak temperature of the resin rises. This may be due to the increased viscosity of the reaction medium, which hinders the fluidity of the reactants and offsets the catalytic effect of YP50S. Therefore, the mass fraction of YP50S used in the experiments is 10 wt%.

**Figure 3:**
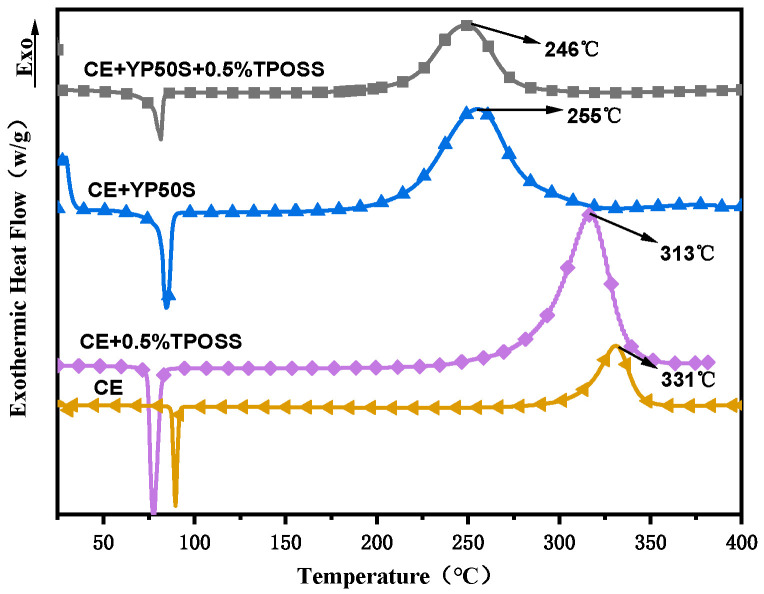
DSC curves of different resin systems.

TPOSS has a significant catalytic (promoting) effect on the curing of the pure CE system, allowing the curing reaction to proceed at a lower temperature. This may be because the rigid cage structure of TPOSS can locally enrich CE molecules, increase the concentration of reaction sites, and accelerate the reaction process. In the YP50S-modified system, TPOSS can further reduce the curing temperature. This is due to the synergistic effect between YP50S and TPOSS. When YP50S and TPOSS coexist, the phenolic hydroxyl groups of YP50S and the polar groups on the surface of TPOSS provide more nucleophilic/proton transfer sites and form a more efficient catalytic network. As can be seen from Figure 3, the combined effect of the two catalysts reduces the reaction activation energy more than that of a single catalyst, leading to a lower curing temperature compared to using YP50S or TPOSS alone.

### 3.2. Rheological Behavior Analysis

**Figure 4:**
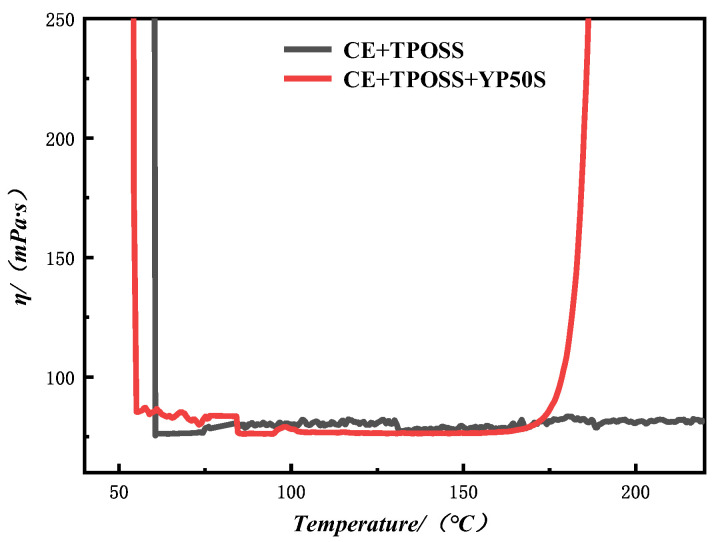
Viscosity-temperature curves of different resin systems.

The performance of the carrier film prepared by the hot-melt method is mainly determined by the rheological properties of the resin system. In the hot calendaring process, it is difficult for resins with too high or too low viscosity to form a good-quality film. Therefore, CE + TPOSS and CE + TPOSS + YP50S film resins were tested using a rheometer. Figure 4 shows the viscosity-temperature curves of the different resin systems.

After adding YP50S, the viscosity of the whole system was high at low temperatures (<50 °C). As the temperature increased to 50–150 °C, the viscosity decreased rapidly and entered a low-viscosity plateau, showing good processing fluidity. When the temperature exceeded about 150 °C, the viscosity of the system increased sharply, indicating the rapid initiation of the curing reaction and the formation of the crosslinking network, which improves the heat resistance and mechanical properties of the substrate. At the same time, the low-viscosity plateau ensures the impregnation of the resin into the basalt fiber and reduces the internal voids of the substrate. From the comparison, it can be seen that the introduction of YP50S significantly reduces the initial curing temperature of the system and achieves rapid crosslinking curing at medium temperature, effectively overcoming the slow curing rate of the pure CE + TPOSS system. This further improves the production efficiency and comprehensive performance of the substrate.

### 3.3. Effect of Different Toughening-Modified Cyanate Ester Resins on the Strength of the Carrier

**Figure 5:**
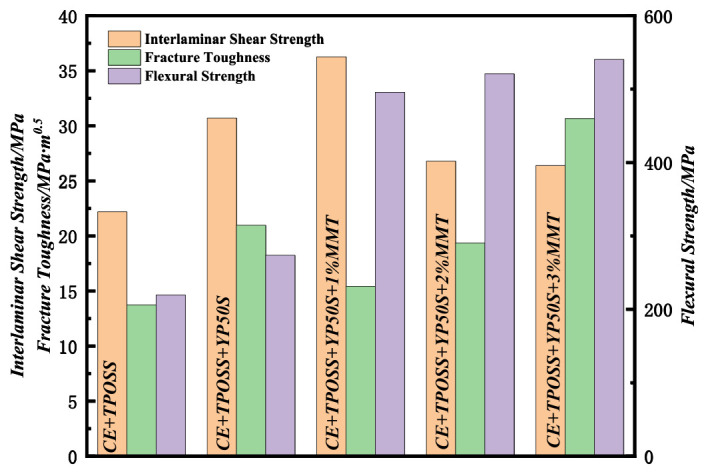
Strength of Resin Carriers with Different Toughening Systems.

As shown in Figure 5 and Table 3, compared with the CE + TPOSS reference system, the introduction of YP50S significantly improved the mechanical properties: interlaminar shear strength increased from 22.18 MPa to 30.70 MPa, fracture toughness from 13.73 MPa·m^0.5^ to 20.96 MPa·m^0.5^, and flexural strength from 219.45 MPa to 273.54 MPa. This enhancement is attributed to the co-curing effect of YP50S with CE, forming a denser crosslinked network, and to hydrogen bonding between phenolic hydroxyl groups and Si–OH on the basalt fiber surface, which strengthens interface adhesion and stress transfer.

Upon incorporation of Si-MMT, the mechanical properties showed a clear content dependence [23]. At 1% Si-MMT, interlaminar shear strength reached a peak of 36.26 MPa, while fracture toughness decreased to 15.41 MPa·m^0.5^ and flexural strength increased to 495.69 MPa. The uniform dispersion of Si-MMT sheets provides a physical barrier and interfacial bonding that enhance shear strength, but the rigid sheets restrict molecular chain mobility, increasing brittleness and reducing toughness.

At 2% Si-MMT, interlaminar shear strength decreased to 26.78 MPa, fracture toughness rebounded to 19.35 MPa·m^0.5^, and flexural strength further increased to 520.75 MPa. Lamellar bridging and crack deflection effects begin to dissipate crack energy, improving toughness, while more rigid layers contribute to load-bearing, resulting in balanced performance suitable for conventional high-performance PCB substrates.

At 3% Si-MMT, interlaminar shear strength stabilized at 26.39 MPa, while fracture toughness and flexural strength reached their peaks at 30.66 MPa·m^0.5^ and 540.49 MPa, respectively. The nanoscale uniform dispersion of Si-MMT (via silicon intercalation modification) enables efficient crack energy dissipation through lamellar bridging and crack deflection. The abundant rigid layers act as a reinforcing phase, synergistically improving toughness and strength. This formulation provides the best overall mechanical performance, meeting the requirements of interlayer bonding reliability, bending resistance, and impact resistance for high-frequency, high-speed, and high-reliability PCB substrates.

### 3.4. Thermal Properties of the Film Resin

The 5% weight loss temperature (t_5_%) was used as an indicator of thermal stability. The t_5_% did not increase monotonically with Si-MMT content; the 2% sample showed the lowest value, while the 3% sample gave the highest. The initial decomposition temperature shifted to higher temperatures only for the 3% sample, whereas the 1% and 2% samples exhibited comparable or slightly lower onset temperatures.

**Figure 6:**
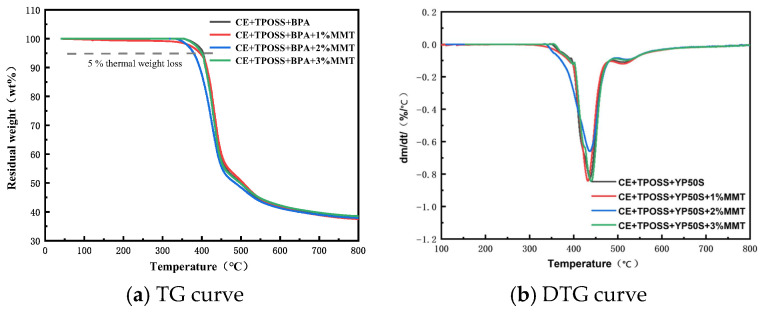
TG and DTG curves of different resin systems.

The DTG curves further reveal that Figure 6 shows the TG and DTG curves of different resin systems (CE + TPOSS + YP50S with 0, 1, 2, and 3 wt% Si-MMT). The overall shapes of the TG and DTG curves are similar, indicating that the addition of YP50S and Si-MMT does not alter the pyrolysis mechanism of the CE resin.

Fect of Si-MMT on thermal decomposition kinetics. For the system without Si-MMT, the maximum decomposition rate is approximately −1.00%/°C at about 420 °C. With the addition of 1%, 2%, and 3% Si-MMT, the peak decomposition rate decreases and shifts to higher temperatures, except that the 2% sample shows a slightly higher rate than the 1% sample. These changes are attributed to the multiple mechanisms of silicon-intercalated Si-MMT.

First, the nanoscale uniformly dispersed Si-MMT sheets form a “maze” physical barrier in the early stage of thermal decomposition, hindering the diffusion and escape of volatile products and delaying heat and oxygen transfer. Second, Si-MMT sheets catalyze the formation of a dense, continuous carbon layer at high temperature, which acts as a thermal and mass barrier, further blocking thermo-oxidative degradation. Third, organic functional groups introduced by silicon intercalation form covalent/hydrogen bonds with the resin matrix, enhancing interface stability and reducing early decomposition caused by interfacial defects. The molecular modeling of such intercalated clay structures has been comprehensively reviewed by Zhou et al. [24], providing theoretical insight into the barrier and interfacial mechanisms.

At 800 °C, the residual mass of all systems is stable at about 40 wt%, with very small differences among them, indicating that Si-MMT has a limited effect on the final char yield. Its core role is to influence the initial decomposition stage and the decomposition rate. Overall, only the 3% Si-MMT modified system clearly improves thermal stability, showing the highest t_5_% (400 °C) and the lowest maximum decomposition rate, which is consistent with the mechanical property optimization. In contrast, the 1% and 2% loadings do not enhance thermal stability; the 2% loading even slightly reduces it, especially in the initial decomposition stage. This non-monotonic behavior may be due to incomplete dispersion or catalytic effects of Si-MMT at lower contents.

**Figure 7:**
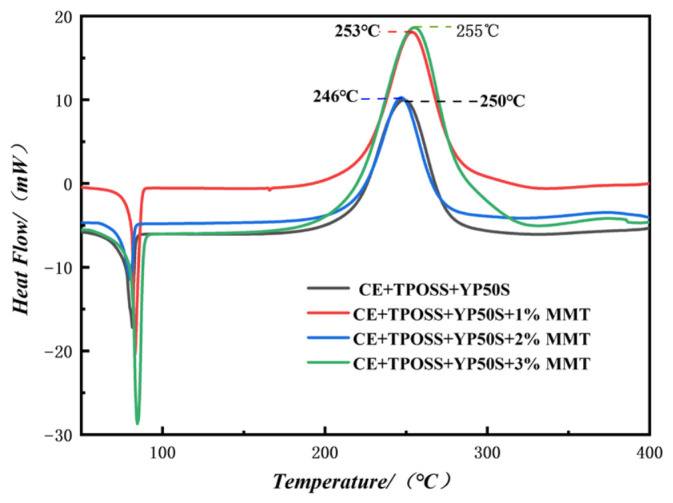
DSC curves of different resin systems.

DSC tests were performed on the cured resins to analyze their curing behavior. Figure 7 shows the DSC curves of the different resin systems [25].

As shown in Figure 7, the curing exothermic peak of the CE + TPOSS + YP50S system without montmorillonite appears at a peak temperature of 250 °C, reflecting the mild curing characteristics under the synergistic catalysis of TPOSS and YP50S. After introducing 1–3% silicon-intercalated montmorillonite (Si-MMT), the curing exothermic peaks shift to different temperatures: 1% Si-MMT gives a peak at 253 °C, 2% Si-MMT gives a peak at 246 °C, and 3% Si-MMT gives a peak at 255 °C. This indicates that the effect of Si-MMT on the curing reaction is content-dependent rather than a simple inhibition. A low amount (1%) slightly increases the peak temperature, a medium amount (2%) slightly decreases it, and a higher amount (3%) again increases it. Therefore, we describe this as a modulating effect of Si-MMT on the curing reaction, which can be attributed to the following mechanisms:

(1) Physical barrier and steric hindrance: Silicon intercalation enlarges the interlayer spacing of montmorillonite, forming a more uniform nanodispersed phase that hinders contact between cyanate ester groups and catalytic sites, and reduces the crosslinking reaction rate.
(2) Adsorption and catalytic weakening: Silane groups and intercalated silicon-oxygen structures adsorb and partially shield the active catalytic sites in TPOSS and YP50S through polar interactions, weakening their synergistic catalytic efficiency and increasing the activation energy of the curing reaction.
(3) Dilution effect: The enlarged interlayer spacing allows some resin molecular chains to insert into the interlayer, partially reducing the concentration of reactive monomers and further delaying the curing process.

From the peak shape evolution, the peak width of the curing exothermic peak for Si-MMT modified systems increases slightly and the peak height decreases, indicating that Si-MMT makes the curing reaction rate more gentle and the reaction process milder. However, all systems eventually complete full crosslinking, as the exothermic curve returns to the baseline. These results demonstrate that silicon-intercalated modified montmorillonite (Si-MMT) can serve as an effective curing regulator, enabling precise control of the curing temperature and reaction rate of the CE + TPOSS + YP50S system. This provides an experimental basis for optimizing the resin processing window and improving process stability.

### 3.5. Dielectric Properties of Carrier Film

**Figure 8:**
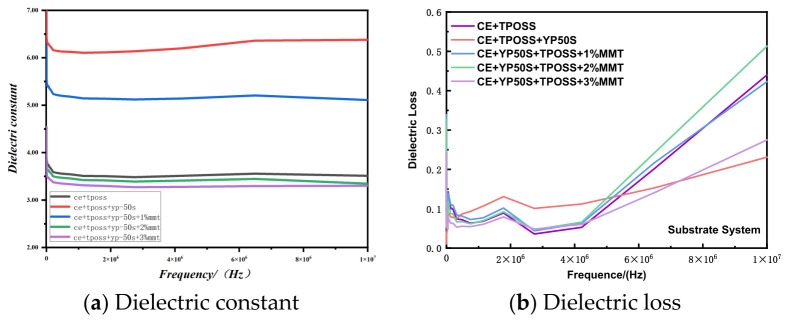
Dielectric constant (**a**) and dielectric loss (**b**) of different resin carrier films at different frequencies. For exact sample compositions, see text in Section 3.5 or Table 1.

The dielectric properties of the carrier film are determined by the matrix resin and the basalt fiber. Basalt fiber has excellent dielectric properties, with a dielectric constant of 2.61 and a dielectric loss of 0.0068 [26]. Figure 8 shows the dielectric constant and dielectric loss curves of the different resin carrier films.

The exact compositions of the samples in Figure 8 are as follows (see also Table 1): CE + TPOSS (CE with 0.5 wt% TPOSS); CE + TPOSS + YP50S (CE with 0.5 wt% TPOSS and 10 wt% YP50S); and CE + TPOSS + YP50S further modified with 1, 2, or 3 wt% Si-MMT (denoted as +1% Si-MMT, +2% Si-MMT, +3% Si-MMT, respectively).

The evolution of dielectric constant and dielectric loss of different resin formulations in the frequency range from 0 to 1 × 10^7^ Hz was systematically studied by broadband dielectric spectroscopy, providing an experimental basis for evaluating their high-frequency electronic application potential [27]. The dielectric constant of each system decreases slowly with increasing frequency, which is a typical dielectric relaxation behavior of polymers: the orientation polarization of polar groups at high frequencies cannot keep up with the alternating rate of the electric field, and the polarization degree is weakened, resulting in a slight decrease in dielectric constant with increasing frequency.

The dielectric constant of the CE + TPOSS system is 3.52 at 1 × 10^7^ Hz. Upon incorporation of YP50S, the dielectric constant decreases to 2.58, indicating that YP50S reduces the overall polarity of the system. When 1–3 wt% of Si-MMT is further introduced, the dielectric constant shows a non-monotonic trend: 1% Si-MMT increases it to 5.11; 2% Si-MMT reduces it to 3.35; and 3% Si-MMT further lowers it to 3.00, which is slightly below that of the reference CE + TPOSS system (3.52). This trend suggests that at low loading (1%), Si-MMT may introduce interfacial polarization or agglomeration that temporarily raises the dielectric constant, while at higher loadings (2–3%), better dispersion and polarity shielding dominate, leading to a decrease.

The dielectric loss (tanδ) increases with increasing frequency. The CE + TPOSS system exhibits a loss of 0.438 at 1 × 10^7^ Hz. After introducing YP50S, the loss decreases to 0.231, indicating reduced energy dissipation. With further addition of Si-MMT, the loss shows a content-dependent behavior: 1% Si-MMT gives a loss of 0.421; 2% Si-MMT gives 0.511; and 3% Si-MMT gives 0.275. The loss at 3% Si-MMT is close to that of the CE + TPOSS system (0.438). These variations may be attributed to competing effects of interfacial polarization, filler dispersion, and polarity shielding.

The synergistic effect of YP50S, TPOSS and Si-MMT can be summarized as follows: YP50S acts primarily as a reactive toughener and curing catalyst through its flexible chains and hydroxyl groups; TPOSS, with its rigid cage structure, locally enriches cyanate ester monomers and provides additional polar sites that further accelerate the curing reaction in cooperation with YP50S; Si-MMT, uniformly dispersed at the nanoscale, creates a physical barrier that delays thermal degradation, reduces the dielectric constant via polarity dilution and shielding, and simultaneously improves mechanical properties through lamellar bridging and crack deflection. The combination of these three components allows the final composite to achieve low curing temperature, high toughness, excellent thermal stability, and low dielectric constant—properties that cannot be obtained by any single modifier alone.

## 4. Conclusions

This study developed a multi-component synergistic modification system for cyanate ester (CE) resin by incorporating YP50S, TPOSS, and silicon-intercalated montmorillonite (Si-MMT) to meet the comprehensive performance requirements of high-frequency and high-speed copper-clad laminate substrates. The effects of these modifiers on curing behavior, rheology, mechanical properties, thermal stability, and dielectric properties were systematically investigated.

The results demonstrate that YP50S effectively promotes the curing reaction and improves the low-temperature fluidity of the resin, while TPOSS further lowers the curing temperature synergistically with YP50S. Si-MMT modulates the curing rate and significantly enhances thermal stability by delaying thermal degradation. Regarding mechanical properties, YP50S improves interlaminar shear strength and toughness, and an optimal amount of Si-MMT (3%) achieves a balance between strengthening and toughening. Dielectric property analysis shows that Si-MMT reduces the dielectric constant while maintaining low dielectric loss, making the system suitable for high-frequency applications.

Overall, the modified CE system containing 10% YP50S, 0.5% TPOSS, and 3% Si-MMT exhibits the best comprehensive performance, including excellent processability, mechanical reliability, heat resistance, and low-loss characteristics at high frequencies. This work provides a theoretical and experimental basis for designing high-performance cyanate ester-based composites for advanced copper-clad laminate substrates.

## Figures and Tables

**Table 1:** Formula of modified cyanate ester resin system unit: %.

Sample	wYP50S	wTPOSS	wSi-MMT
CE-0	0	0.5	0
CE-Y5	5	0	0
CE-Y10	10	0	0
CE-Y15	15	0	0
CE-Y-T10	10	0.5	0
CE-Y-T10-M1	10	0.5	1
CE-Y-T10-M2	10	0.5	2
CE-Y-T10-M3	10	0.5	3

Note: The contrast sample ‘CE−0’ was obtained by melting the unmodified cyanate ester resin at 120 °C and adding a small amount of catalyst TPOSS.

**Table 2:** Exothermic peak temperatures of the blends with different mass fractions of YP50S and CE.

Sample	Exothermic Peak/°C
CE-0	331
CE-5%YP50S	274
CE-10%YP50S	242
CE-15%YP50S	244

**Table 3:** Mechanical properties of different resin carrier films (mean ± SD, *n* = 5).

Sample	Interlaminar Shear Strength (MPa)	Fracture Toughness (MPa·m^0.5^)	Flexural Strength (MPa)
CE + TPOSS	22.18 ± 1.10	13.73 ± 0.70	219.45 ± 11.00
CE + TPOSS + YP50S	30.70 ± 1.50	20.96 ± 1.00	273.54 ± 13.70
CE + TPOSS + YP50S + 1%Si-MMT	36.26 ± 1.80	15.41 ± 0.77	495.69 ± 24.80
CE + TPOSS + YP50S + 2%Si-MMT	26.78 ± 1.30	19.35 ± 1.00	520.75 ± 26.00
CE + TPOSS + YP50S + 3%Si-MMT	26.39 ± 1.30	30.66 ± 1.50	540.49 ± 27.00

## Data Availability

The original contributions presented in this study are included in the article. Further inquiries can be directed to the corresponding author.

## References

[1] Hu J., Xu Y., Hu X. Research and Application of Heat-Resistant and Low-Loss Acenaphthylene Copolymer Resin. Proceedings of the 25th China Copper Clad Laminate Technology Seminar.

[2] Shen M., Qin H. (2024). Study on a Cyanate Ester/Benzoxazine/Epoxy System for High-Frequency Copper Clad Laminates. Thermosetting Resin.

[3] Wang L., Yang J., Cheng W., Zou J., Zhao D. (2021). Progress on Polymer Composites with Low Dielectric Constant and Low Dielectric Loss for High-Frequency Signal Transmission. Front. Mater..

[4] Dong J., Wang H., Zhang Q., Yang H., Cheng J., Xia Z. (2022). Hydrocarbon Resin-Based Composites with Low Thermal Expansion Coefficient and Dielectric Loss for High-Frequency Copper Clad Laminates. Polymers.

[5] Wang D., Hou D., Chen Z., Ma H., Huang C., Yang L. (2020). Effects of Trace Phenolic Hydroxyl Groups on the Cure Behaviours and Properties of Cyanate Esters. High Perform. Polym..

[6] Ma Q., Lin F., Liu X., Wang W., Lu Z. (2025). Toughening and High-Temperature Modification of Cyanate Ester Resin. Fiber Compos..

[7] Li X., Liu X., Feng J., He R., Liu H., Hu X., Liu X. (2023). A research on benzoxazine/cyanate ester/epoxy POSS nanocomposite with low dielectric constant and improved toughness. Polym. Bull..

[8] Goyal S., Cochran E.W. (2022). Cyanate Ester Composites to Improve Thermal Performance: A Review. Polym. Int..

[9] Zhou J., Jin C., Wang F., Zhu Y., Qi H. (2025). Mechanism and Properties of the Room-Temperature Catalyzed Curing of a Cyanate Ester/Epoxy Resin System by Aromatic Amines. ChemistrySelect.

[10] Ren C., Liu F., Zhu H. Preparation and Properties of Special Phenoxy Resin for Copper Clad Laminates. Proceedings of the 25th China Copper Clad Laminate Technology Seminar.

[11] Ren C., Liu F., Zhu H. A Special Phenoxy Resin Applicable to Package Substrates. Proceedings of the 26th China Copper Clad Laminate Technology Seminar.

[12] Reddy A.M., Kandasubramanian B., Rath S.K. (2023). Cyanate ester blends and composites to improve dielectric, mechanical, and thermal performance for functional applications. Polym. Bull..

[13] Örçen G., Bayram D. (2024). Effect of Nanoclay on the Mechanical and Thermal Properties of Glass Fiber-Reinforced Epoxy Composites. J. Mater. Sci..

[14] Kök M., Bulut A., Tayfun Ü. (2025). The Efficiency of Isocyanate Coating on the Basalt Fibre Surface for the Mechanical Performance of Elastomeric Polyurethane Composites. J. Elastomers Plast..

[15] Guenthner A.J., Sahagun C.M., Lamison K.R., Reams J.T., Haddad T.S., Mabry J.M. (2015). Effect of Nanoparticle Functionalization on the Performance of Polycyanurate/Silica Nanocomposites. Ind. Eng. Chem. Res..

[16] Ganguly S., Das P., Das T.K., Ghosh S., Das S., Bose M., Das N.C. (2020). Acoustic cavitation assisted destratified clay tactoid reinforced in situ elastomer-mimetic semi-IPN hydrogel for catalytic and bactericidal application. Ultrason. Sonochem..

[17] Feher F.J., Newman D.A., Walzer J.F. (1989). Silsesquioxanes as models for silica surfaces. J. Am. Chem. Soc..

[18] Jiang J., Tao X., Yang T., Chen D., Yin C., Xing S., Tang J. (2024). Synergistic improvements in the processability and mechanical properties of cyanate esters via aminobenzonitrile-induced chemical tailoring. J. Mater. Chem. A.

[19] Sobczak J., Cioch K., Żyła G. (2025). Paraffin-Based Composites Containing High Density Particles: Lead and Bismuth and Its Oxides as γ-Ray Shielding Materials: An Experimental Study. Discov. Nano.

[20] Han B., Li Y., Wan J., Hu W., Chu Q., Shi Y., Yang L., Hu Z. (2025). Progress in High Temperature Resistant Phthalonitrile Resins and Their Composites for Aerospace Applications. React. Funct. Polym..

[21] Galukhin A., Nosov R., Taimova G., Shulyatiev A., Nikolaev I., Islamov D., Vyazovkin S. (2022). Mechanistic and kinetic insights into phenol-catalyzed cyclotrimerization of cyanate esters. Thermochim. Acta.

[22] Remiro P.M., de la Caba K., Mondragon I., Riccardi C.C. (2010). Influence of phenoxy addition on the curing kinetics for uncatalyzed and catalyzed cyanate ester resin. J. Appl. Polym. Sci..

[23] Liu H., Sun Y., Yu Y., Zhang M., Li L., Ma L. (2022). Effect of Nano-SiO_2_ Modification on Mechanical and Insulation Properties of Basalt Fiber Reinforced Composites. Polymers.

[24] Zhou A., Du J., Zaoui A., Sekkal W., Sahimi M. (2025). Molecular modeling of clay minerals: A thirty-year journey and future perspectives. Coord. Chem. Rev..

[25] Mu M., Vaughan A. (2021). Dielectric Behaviours of Bio-Derived Epoxy Resins from Cashew Nutshell Liquid. High Volt..

[26] Li Z., Ma J., Ma H., Xu X. (2018). Properties and Applications of Basalt Fiber and Its Composites. IOP Conf. Ser. Earth Environ. Sci..

[27] Ren J.W., Jiang G.Q., Chen Z.J., Wei H.C., Zhao L.H., Jia S.L. (2024). Surface Structure Design of Boron Nitride Nanotubes and the Mechanism of Their Regulation of the Properties of Epoxy Composite Dielectrics. Acta Phys. Sin..

